# A Method and Device for Automated Grinding of Small Ceramic Elements

**DOI:** 10.3390/ma14247904

**Published:** 2021-12-20

**Authors:** Wojciech Kacalak, Dariusz Lipiński, Filip Szafraniec, Błażej Bałasz

**Affiliations:** Faculty of Mechanical Engineering, Koszalin University of Technology, Racławicka 15, 75-620 Koszalin, Poland; wojciech.kacalak@tu.koszalin.pl (W.K.); blazej.balasz@tu.koszalin.pl (B.B.)

**Keywords:** grinding, ceramics, optimization, modeling, automation

## Abstract

The paper describes an automated method for grinding small ceramic elements using a hyperboloid wheel. The problem of automating the process of machining elements made of nonmagnetic materials with a small area and low height has been solved. Automation of the grinding process was possible thanks to automatic clamping of workpieces in the machining zone and sequential processing by a specified number of grinding wheels. The workpieces were passed through successive machining zones. The division of the allowance of individual grinding wheels was made taking into account the characteristics of the workpieces and the requirements for the results of the machining. Obtaining a long grinding zone and the effect of automatic clamping of the workpieces was possible due to the inclination of the grinding wheel axis in relation to the plane of movement of the workpieces. Innovative aggregate grinding wheels were used for grinding. The aggregates containing diamond abrasive grains, connected with a metal bond, were embedded in the porous structure of the resin bond. The aggregates ensured high efficiency of grinding, and their developed surface contributed to good holding in the resin binder. The durability of grinding wheels was 64 h, which enables the machining of 76,000 ceramic elements.

## 1. Introduction

Technical ceramics are materials that are increasingly used in many fields, such as in electronics, cutting tools, technological devices, optics, measuring equipment, propulsion systems, medicine, space and military technology, and microengineering. Ceramic materials, depending on the composition and production technology, have various favorable properties, and above all, they are characterized by high hardness, stable piezoelectric properties, stable relative magnetic permeability, fast ion conduction, high magnetic permeability, possible optical transparency, high radiation resistance. Moreover, in many applications, important features of ceramics are their high melting point, high mechanical strength at high temperatures, and high abrasive wear resistance. This results in further advantages: high resistance to chemical influences, high shape stability, creep resistance, good thermal insulation, fairly good mass-to-volume ratio.

Many of the listed properties adversely affect the workability of the ceramics. This problem occurs especially when high accuracy after machining and very low surface roughness is required from the parts being manufactured. A characteristic feature that clearly distinguishes ceramic materials from metal materials is their low ductility and low fracture toughness regardless of strength. In ceramics, grinding processes, high-quality machining is possible when the energy consumption of the process is reduced and the gradient of mechanical and thermal load on the workpiece surface in the grinding zone is reduced [1,2,3,4]. This means limiting the formation and propagation of cracks especially near the edge of the workpiece [5].

The increase in the use of technical ceramics and composites is mainly influenced by the needs of strategic industries, military applications, the development of electronics, and mechatronics. Requirements for reliability and high durability as well as social considerations related to health protection and environmental protection are becoming more and more important [6]. Ceramic materials subjected to abrasive treatment differ significantly in many features, such as chemical composition, hardness, internal structure, brittleness, and porosity [5,7,8,9,10,11].

The most common abrasive machining method for oxide ceramics and oxygen-free ceramics is grinding with diamond grinding wheels. It is accompanied by significant forces and large temperature gradients. Conventional precision grinding processes are not suitable for microgrinding and polishing operations on ceramics. In typical grinding processes, the grinding depth is many times greater than the surface roughness height before machining. Shape deviations, especially for small-sized workpieces, are also much smaller than the grinding depths used.

Robotized machining centers can be used for finishing, especially of large elements with complex geometry [12]. For the implementation of ceramics grinding processes, among others, specialized multiaxis microgrinding centers [13,14,15] are built and their dynamic characteristics are improved [16]. In the case of machining small ceramic elements, the main technological problem is the automation of the process. Providing high-performance grinding of components made of nonmagnetic materials is difficult in several cases. One of them occurs when the elements do not have a surface area greater than 2–3 cm^2^ needed for vacuum fastening. The second case is when the workpieces are of low height, e.g., less than 1 mm. The method of fixing small elements by sticking them on and then processing them used in such cases does not meet the conditions of automation and the required efficiency. In such situations, there is usually only the possibility of using a specially shaped table surface and the clamping effect of the grinding force, which was the main assumption for the developed method and the automatic grinders built. The required high processing efficiency can only be ensured by the use of fully automated feeding, clamping, and unloading of objects and the processing method ensuring the removal of the stock in one pass. This is possible through the use of a processing method that provides a long grinding zone with a low infeed and a low stock removal rate along the entire length of this zone.

The analysis of the precise processing of ceramic materials covered the problems of dimensional accuracy and flatness of the surface as well as the geometric structure of the surface [17,18]. It also concerned the optimization of the relationship between machining forces and the strength of workpieces of very small heights. An important criterion for the machining quality was the geometric edge accuracy and the surface microstructure. It was found that meeting these requirements was possible through the use of a new method and new tools. Conclusions came from the resent investigations on the features of abrasive tools [3,8,19,20], process kinematics [21], the influence of the processed material properties on the process results [22,23,24,25,26] and cutting fluid [9,27]. In the assumptions for the new method, the developed methodology of process monitoring [28,29], a thorough analysis of the properties of abrasive tools [30,31,32], and the topography of the treated surfaces [33] were used. The results of the simulations described in the works [34,35] were used into account which showed that in the recent period of application of grinding processes, they even extended to the nanometric scale. The simulations were very helpful in the study of various phenomena related to micro- and nanogrinding [36], as the direct determination of local values of many process features is limited.

In the case of grinding ceramics with the use of diamond grinding wheels, the efficiency and quality of machining depend on the relationship between the abrasive wear of individual abrasive grains and the bond strength of these grains with the bond. In general, it is quite difficult to obtain a sufficiently strong bond, especially in the case of binders with the required flexibility. To ensure high machining efficiency and the required durability of the tool, innovative grinding wheels with an aggregate structure were used. The advantages of such tools were described in [1]. Aggregates containing diamond abrasive grains connected with a metal binder were embedded in the porous structure of the resin binder. The aggregates ensured the high grinding efficiency of ceramics, and their developed surface contributed to good holding in the resin binder. The high porosity and favorable flexibility of the resin bond advantageously reduced the local variation of forces in the grinding zone. In conventional grinding methods, the requirements for high machining efficiency and accuracy are met by gradually moving the workpiece repeatedly into the machining zone. The adopted assumption of automation meant that the entire allowance is removed as a result of a single, sequential movement of the workpiece through the grinding zones.

The article presents a new grinding method that ensures high flatness of the treated surface, high dimensional and shape stability of the grinding wheel, and a long grinding zone, in which the speed of removal of the material is slightly reduced. Renewing the hyperboloidal shape of the active surface of the grinding wheel takes place by shaping its lowermost generatrix, which is a horizontal straight segment. In the developed method, the size of the allowance to be removed decreases as the object moves along the zone in which it is ground. The workpieces are processed in one operation by consecutive grinding wheels, with an optimized distribution of the allowance [37]. In the machining zone with the last grinding wheel, the workpieces can be lifted by a dozen or so micrometers and pressed with the air stream to the grinding wheel surface.

The development of a method with such advantageous features required solving many problems of full automatization of feeding, orienting, clamping, removing objects as well as effectively removing processing products from the table surface. This method has been implemented for the production of small ceramic components used in electronic circuits and for the processing of small piezoceramic components.

## 2. Materials and Methods

### 2.1. Purpose and Scope of Research

**Figure 1:**
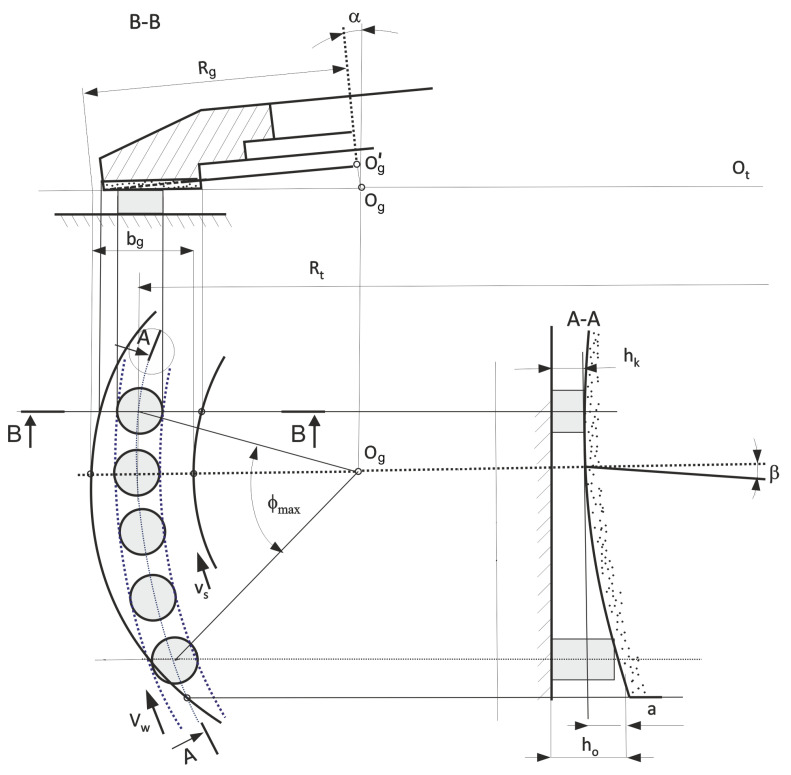
Diagram for the method of face grinding with a grinding wheel with a hyperboloid active surface.

**Figure 2:**
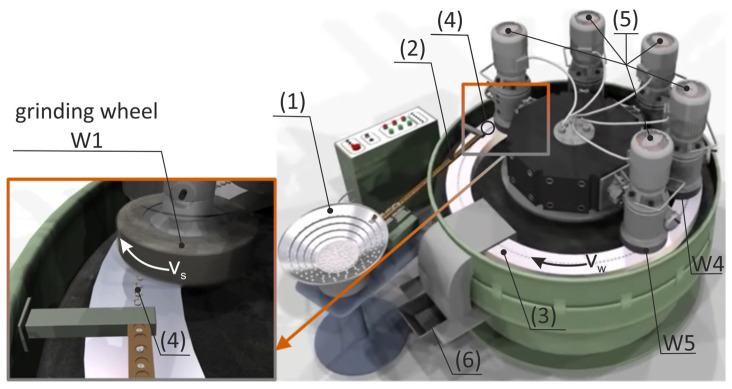
Structural system of an automatic grinder for small ceramic elements (**1**) vibrating spiral feeder, (**2**) vibrating linear feeder, (**3**) rotary table, (**4**) workpieces, (**5**) headstocks, (**6**) container of finish objects.

**Figure 3:**
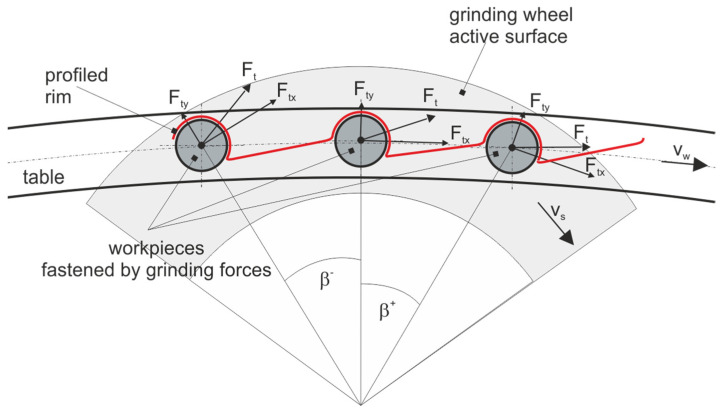
Diagram of clamping the workpieces with grinding forces using the profiled edge of the table.

**Figure 4:**
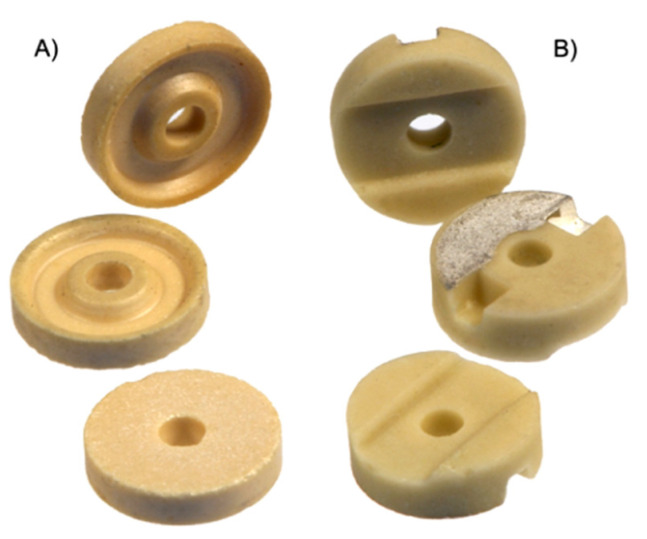
Shape and dimensions of the processed elements (**A**)—rotor, (**B**)—stator.

**Figure 5:**
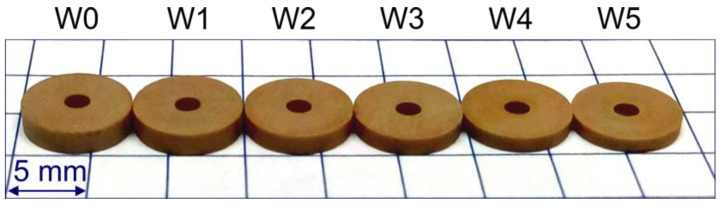
Ceramic elements after grinding in one operation by successive grinding wheels W1–W5 (W0—means state before grinding).

The research aimed to develop a method allowing to reduce the speed of material removal and extend the grinding zone without reducing the grinding efficiency. The assumptions were defined as follows:

1. The use of a very small thickness of the layers of material removed with individual grains allows to significantly reduce the local values of the process energy, which allows more favorable features of the surface layer after treatment to be obtained.
2. The small depth of grinding results in a low density of heat fluxes, a short time of their local impact, and small temperature gradients. The depth of heat penetration into the surface layer of the object is significantly reduced.
3. The use of small layers of material removal reduces grinding forces, which is important for machining small-size and high compliance workpieces or workpieces with low thickness and low pressure resistance.
4. The use of low material removal rates does reduce the proportion of active grains, but it is compensated by the considerable elongation of the grinding zone resulting from the features of the method.
5. Automated grinding of small ceramics can be competitive with lapping processes by increasing productivity, eliminating stacking of items on lapping plates, increasing production flexibility, and extending process monitoring.
6. To obtain a long grinding zone and a decreasing speed of stock removal during the movement of the object under the active surface of the grinding wheel, it was assumed that:
  - The processed elements will move on a circular path with radius *R_t_* (Figure 1) going from the feeding zone through successive processing zones to the zone of placing the finished objects in the container (Figure 2).
  - The machining will be carried out on an angular path ϕ*_max_* with the use of grinding wheels with radius *R_g_* and with a hyperboloid active surface, and the lowest-lying forming (cross-section A-A) will be horizontal (cross-section B-B). The axis of the grinding wheel deviates from the plane of movement of the objects by angles α and β (Figure 1).
  - The machined ceramic elements of low height (*h_o_*) and allowance *a* (Figure 1) will be held on the table by grinding forces as a result of placing the profiled rim in the cutouts (Figure 3).
  - The structural system of the automatic grinder will ensure the processing of objects of various shapes and dimensions (Figure 4 and Figure 5), as a result of changing the periphery.
7. The machining cycle consists of a single pass of the ground elements through several treatment zones.
8. In the grinding process, up to five grinding wheels (Figure 2) with diamond grains, joined by a metal bond to form aggregates with dimensions of approx. 500 µm, will be used. Due to the developed surface, the aggregates were placed in resin binders ensuring high porosity. As a result, the bonding forces of the grains are high, the porosity is good, and the low temperatures in the production of grinding wheels do not limit the introduction of active additives into the volume of the abrasive tools.
9. The allowance removed by subsequent grinding wheels has been divided taking into account the safe relationship between the pressure force on the workpiece and its compressive strength.

The feeding of the objects is carried out by a vibrating spiral feeder with a rejection system of misoriented objects. Then the items go to a vibrating linear feeder with a speed controller. A table with a vertical axis of rotation has evenly spaced open seats formed by a suitably profiled rim. Objects are clamped under the influence of grinding forces. The shape of the rim (red line on Figure 3) ensures a stable position of the workpieces along the entire length of the grinding zone. During the rotation of the table, the workpieces move successively under five headstocks, the diamond grinding wheels of which remove a certain part of the allowance. In addition, the machining system includes a receiving unit, a reservoir, a device for washing and drying the surface of the table, and a system for cooling and cleaning the surfaces of the grinding wheels.

### 2.2. Characteristics of Processed Elements As Well As Parameters and Processing Conditions

The experimental studies used sets of small ceramic elements (Figure 5) machined in the process of testing automated grinders specialized for planes. The tests were carried out for the following parameters: grinding speed *v_s_* = 30 m/s and table rotational speed *v_w_* = 20 mm/s. The ground elements had a diameter of ϕ = 7.4 mm and an initial height *h_0_* = 1.7 ± 0.1 mm and were made of ceramic material (Table 1), the hardness of which was 8 on the Mohs scale.

**Figure 6:**
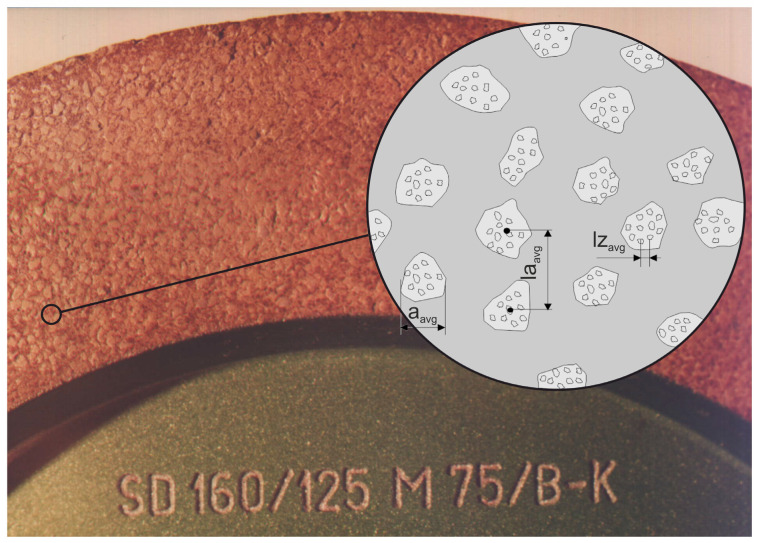
Construction of diamond grinding wheels used in the grinding process of ceramic components.

The height of the elements after the machining process (rotor) was *h_k_* = 0.8–0.01 mm. Due to the shape of the workpieces and a large allowance (0.72 mm), five resin-bonded diamond grinding wheels with a radius of *R_g_* = 100 mm were used for machining (Figure 6, Table 2 and Table 3), mounted on the spindles located around the perimeter of the track of items.

Grinding wheels with the designation W1–W4 worked in the technological system with the axis of the headstock inclined by the angle α and β and their active surfaces were in the form of a hyperboloid. The task of the W5 grinding wheel was to smooth the ground elements and it worked in a typical kinematic system of grinding with the grinding wheel face (α = 0), and its active surface was flat. The values of the α angles for the W1–W5 grinding wheels are presented in Table 4.

The second type of test items were ceramic sealing plates for valves, used, inter alia, for water batteries. The diameter of the plates was 15.8 mm and the height was 3.8 mm. The plates are made of oxide ceramics, alumina with 92–99% Al_2_O_3_ content. Properties of ceramic plates used in sealing rings were as follows: apparent density 3.4–3.8 g/cm^3^, bending strength 250–300 MPa, impact strength 4 kJ/m^2^, Young’s modulus 220–300 GPa, dielectric strength 15–17 kV/mm, resistivity 10^14^ Ω cm, resistance to temperature changes 140–150 K.

### 2.3. Research Stand

**Figure 7:**
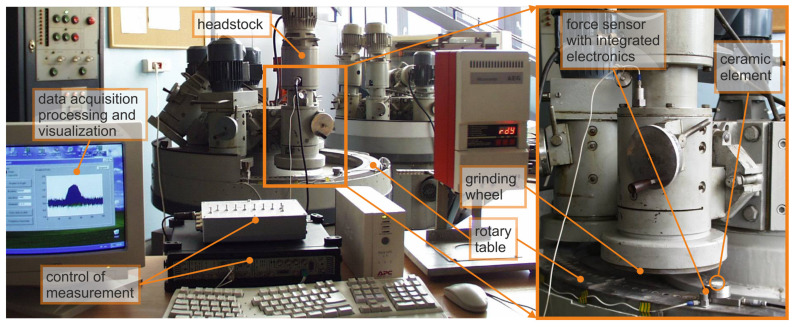
View of the test stand.

The research on the new method of microgrinding of planes with the use of grinding wheels with a hyperboloidal active surface was carried out on a test stand (Figure 7). In the grinding process, the length of the grinding zone for items with a diameter of 7.4 mm was 98 mm and was about five times larger than the width of the active surface of the grinding wheel. In the developed method, apart from the large length of the grinding zone, favorable cross-graining of the grains is obtained. Another advantage is the reduced stock removal speed along the treatment zone. This ensures a favorable surface topography and limits damage to the edges of the workpieces. The active surfaces of the grinding wheels are hyperboloids (in a special case they may be conical, but then the length of the grinding zone is doubled). Shaping the surface of the grinding wheel is not difficult because the lowermost straight line generating the hyperboloids is parallel to the nominal surface of the workpieces. The axis of the grinding wheel is then not perpendicular to the plane of movement of the workpieces but it is inclined in two planes. The angles of the axis of the grinding wheels and the adopted length of the grinding zone affect the parameters of the hyperboloid forming the active surface of the tool.

S3020 200 × 85 × 30 × 4 SD 125/100 BT type resin bond diamond grinding wheels were used to test the grinding phenomena. Grinding parameters *v_s_* = 29.5 m/s. Ceramic elements were ground in the allowance range *a* = 50–200 µm with the table speed *v_w_* = 4–7 mm/s. Water cooling was used.

The values measured in the grinding process were: grinding force components with the use of a three-component Kistler sensor with a measuring range up to 150 N, vibration acceleration of the rotary table, and vibration acceleration of the headstock assembly using the PULSE vibration and acceleration measurement kit by Bauer and Kjear. Elements of the test stand are listed in Table 5.

### 2.4. Methodology of Optimizing the Division of the Allowance in the Process of Sequential Grinding of Small Ceramic Elements

The abrasive machining operation as a final operation throughout the machining process determines the final performance properties of the product. The accuracy of dimensions and shapes plays an important role in the case of the pairing of sliding, rolling, or resting elements, ensuring that the appropriate clearance, interference, or tightness of the connection is obtained.

**Figure 8:**
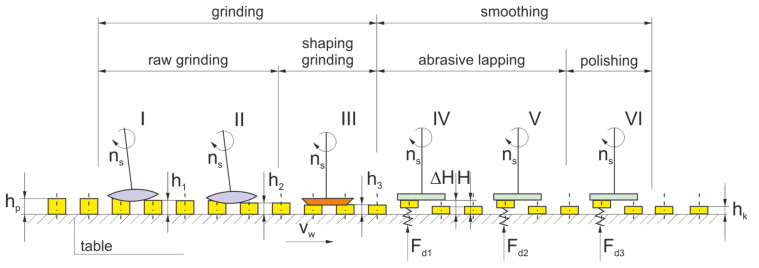
Diagram of the method of integrated processing of ceramic elements in an automatic cycle.

Due to deformations in the processes of shaping and sintering ceramic materials, the grinding allowances are usually quite large. In the sequential machining method, the total stock to be removed was divided between several grinding wheels (Figure 8). The orientation of the axes of individual grinding wheels, the shape of their active surface, and the characteristics of subsequent grinding wheels, depending on it, are varied. The number (1–4) of grinding wheels with an axis inclined towards α and β depends on the type and dimensions of the workpieces. The number of smoothing and polishing wheels can be 1–3.

The elements, placed in the open (Figure 8) seats of the rotary table, are successively processed by aggregate diamond grinding wheels with smaller and smaller grains and decreasing hardness. They are then shaped and smoothed by grinding wheels containing microdiamond grains. During roughing and shaping, the elements are fixed on the surface of the turntable, and during smoothing, they can be lifted and flexibly pressed against the surface of the tool by means of a pneumatic diaphragm. In the machining method used, the elements move under the grinding wheel, rotating in the direction of movement of the workpieces (downgrinding), along a circular path with a large radius.

**Figure 9:**
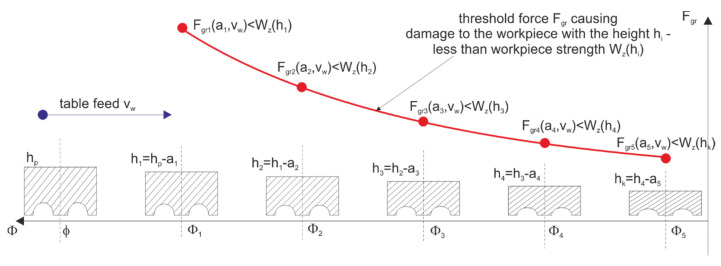
Change in the strength of workpieces as a result of reducing their height while passing through successive machining zones.

The quality and efficiency of the process depend on the division of the allowance on individual grinding wheels and the selection of the table feed speed. Machine, workpiece, and grinding wheel constraints affect the optimization result. In the described processes of grinding ceramic elements, significant limitations include: limit dimensional deviations, limit values of roughness parameters and surface quality, the resistance of the machined elements to loading with machining forces, and equal grinding wheel life. Since in the considered grinding process the dimensions of the elements and the grinding power required to remove the stock are small, there is no limitation related to the power of the machine tool. The main limitation, however, is the strength of the items, which additionally decreases along with the reduction of their height during the removal of successive layers of the allowance (Figure 9).

Low strength of objects (resulting from their shape) limits the permissible value of the normal grinding force component, and thus also the machining efficiency.

To optimize the division of the allowance, the criterion was the highest volumetric grinding efficiency *Q_v_* μm^3^/s in a given machining cycle. The total volumetric efficiency of the process is the sum of the performance of all grinding wheels used in the machining, each of which is designed to remove a layer of material with a thickness of *a_i_*.

(1) $$Q_{v}=\sum_{i=1}^{N}Q_{vi}=A\cdot \frac{v_{w}}{T_{o}}\cdot \sum_{i=1}^{N}a_{i},$$

where: *A*—area of the processed surface, *T_o_*—the peripheral scale of the arrangement of elements on the rotary table of the grinder.

Workpiece speed is a process controllable parameter. The limiting speed of the movement of the workpieces depends on the division of the allowance into individual grinding wheels, so that none of them limits the speed to a greater degree than the others. The optimal division of the allowance is therefore the division for which the permissible feed rate is the highest. This means maximizing process performance when constraints are met.

So, the task of optimizing the division of the allowance comes down to determining the process parameters $p_{obr}=\left\{a_{1},\ldots ,a_{N},v_{w}\right\}\;$ determining the amount of allowance removed by individual grinding wheels and the table speed for the optimization criterion, which is the machining efficiency $Q_{vwym}$. It is connected with the fulfillment of the requirements concerning the limitation of the normal grinding force due to the strength of the processed elements.

By allowing the value α of the probability *$p\left(h_{i}|F_{ni}\right)$* of damage to objects with a variable height *h_i_*, loaded with the normal component of the grinding force *F_ni_*, resulting from the speed *v_w_* and the thickness of the allowance removed on the *i*-th headstock, a set of optimal machining parameters *p_obr_* can be determined as follows:

(2) $$\underset{p_{obr}\in P}{\forall }\left(Q_{v}=Q_{vwym}\wedge \underset{i\in 1..N}{\exists }p\left(h_{i}|F_{ni}\right)\leq \alpha \right),$$

**Figure 10:**
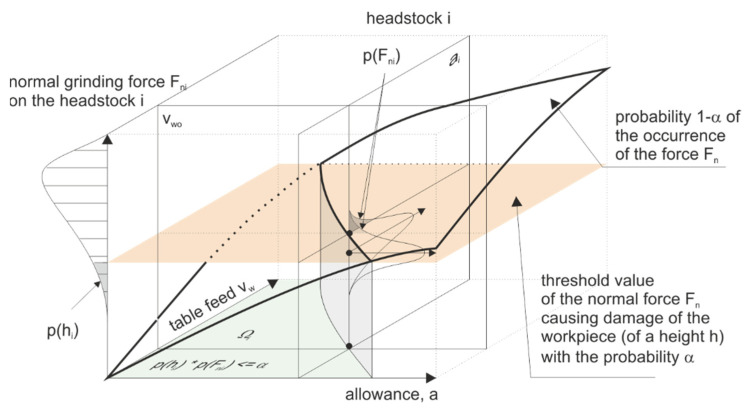
Selection of machining parameters on the *i*-th headstock ensuring technological safety of the process.

A graphic presentation of the above assumptions is shown in the figure (Figure 10).

The knowledge of the limiting value of the normal grinding force *F_ngri_* component makes it possible to determine for each of the *N* headstocks a set of parameters $\Omega_{i}\in \left\{a_{i},v_{w}\right\}\;$ such that:

(3) $$\Omega_{i}:\underset{a_{i},v_{w}\in P}{\forall }F_{ni}\left(a_{i},v_{w}\right)\leq F_{ngri},$$

For this set of parameters it is possible to determine allowances *a_i_* removed by successive headstocks satisfying the condition:

(4) $$\overset{N}{\underset{i=1}{\sum }}a_{i}=a_{c},$$

where: *a_c_*—the total amount of the allowance to be removed.

There are three strategies for dividing the allowance and its impact on technological security and efficiency of the machining process.

**Figure 11:**
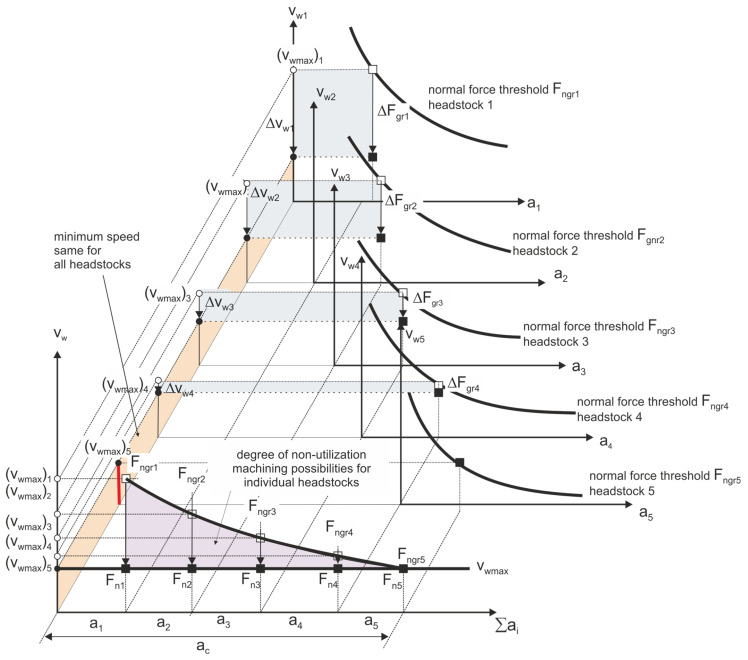
Set of machining parameters for individual headstocks ensuring the required machining efficiency while maintaining an even division of the machining allowance—Strategy 1.

Strategy 1. Uniform distribution of the allowance—the machining allowance is evenly distributed over all grinding wheels sequentially participating in the machining, which are characterized by increasingly smaller grain sizes (Figure 11).

The assumed strategy of optimizing the process efficiency, while maintaining its technological safety, leads to the selection of the table speed according to the dependence:

(5) $$\min\left({\left(v_{wmax}\right)}_{i}\right),$$

The maximum machining efficiency is significantly limited by the maximum value of the allowable speed on one of the headstocks. The use of such a process parameters optimization strategy leads to a situation in which the machining capabilities of only one of the headstocks are used to the maximum extent.

**Figure 12:**
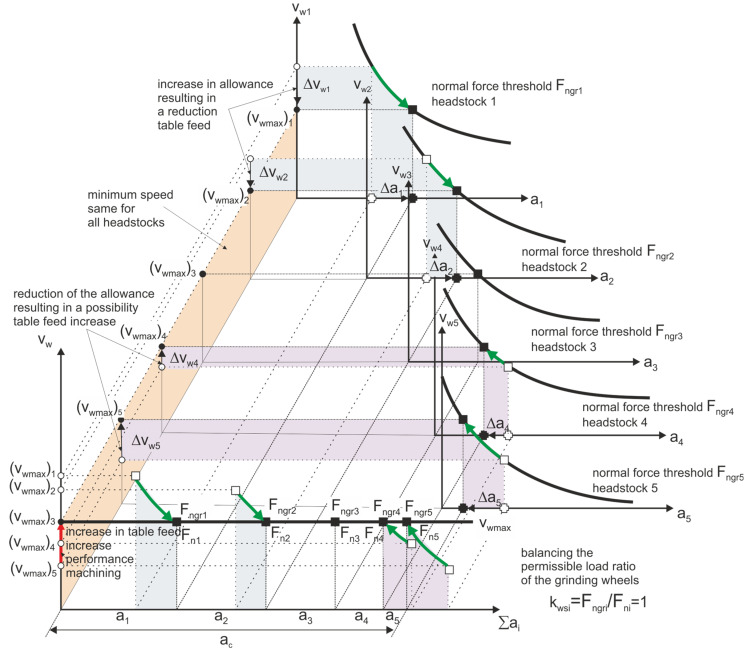
Set of machining parameters for individual headstocks ensuring the required machining efficiency while maintaining the optimal division of machining allowance—Strategy 2.

Strategy 2. Optimal allocation of allowance—constant load-to-limit ratio. The machining allowance is distributed over the grinding wheels with the assumption of maximizing the allowable wheel load factor *k_ws_*. This coefficient is the ratio of the normal grinding force *F_n_* component to the normal grinding force *F_ngr_* component causing damage to the workpiece with a probability equal to α. (Figure 12).

In the assumed optimization strategy, such a division of the allowance was sought that would ensure the highest value of the rotary table traverse speed $v_{wmax}$ ensuring maximum machining efficiency. A new set of allowance values was obtained $\Omega \underset{v_{wmax}\underset{i\in 1..N}{\exists }\underset{a_{i}\in \Omega }{\forall }\sum a_{i}=a_{c}}{\forall }$ on individual headstocks. By selecting the size of allowances for individual grinding wheels, under the objective function specified below:

(6) $$\max\left(\min\left(v_{max}{\left(a_{i}\right)}_{i}\right)\right),$$

the permissible loads of individual grinding wheels were equalized $\;k_{wsi}=F_{ni}/F_{ngri}=1$, thus increasing the table speed and thus the machining efficiency.

**Figure 13:**
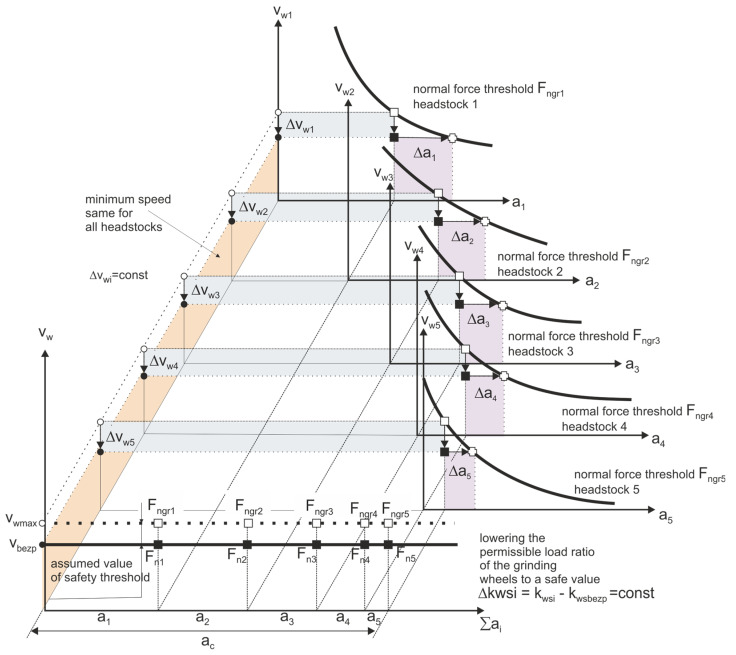
Set of machining parameters for individual headstocks ensuring the required machining efficiency while maintaining the optimal division of the machining allowance, taking into account the safe load threshold of the grinding wheels—Strategy 3.

Strategy 3. Optimal division of allowance—constant difference between the decreasing strength of elements and their load with normal grinding force. The adopted machining allowance on individual grinding wheels ensures a constant workpiece safety threshold *k_wsi_* = *k_wsi_* − *k_wsafe_* = const (Figure 13).

This strategy assumes lowering the speed *v_w_* of the workpieces to a value that ensures a constant safety margin of the permissible grinding wheel load. It allows the possibility of an increase in the normal grinding force component as a result of inaccuracies in the process without the risk of a defective product appearing as a result of machining.

Examples of optimization results leading to the determination of safe parameters of the machining process allowing an increase in the monitored features of the process by 20% of the limit value, are presented in Table 6.

A gradual change in the properties of grinding wheels during their operation life, leads to an increase in grinding power and forces, which affects the disturbance of the criterion of constant safety on individual headstocks. Therefore, further machining requires the determination of a new division of the allowance for individual headstocks while maintaining the assumed machining efficiency and technological safety of the process.

Compensation of changes in the properties of grinding wheels during their operation life by changing the division of the allowance for individual grinding wheels is possible only within a limited range of parameters resulting from the adopted speed of the rotary table *v_w_*. If the machining efficiency is higher than required, it is possible to reduce the speed of the rotary table to the speed *v_wmin_* that meets the minimum requirements for the assumed performance criteria. Then, the time after which it becomes necessary to renew the cutting properties of the grinding wheel can be defined as the moment of reducing the performance to the level equal to the minimum allowable volumetric performance *Q_vmin_*.

**Figure 14:**
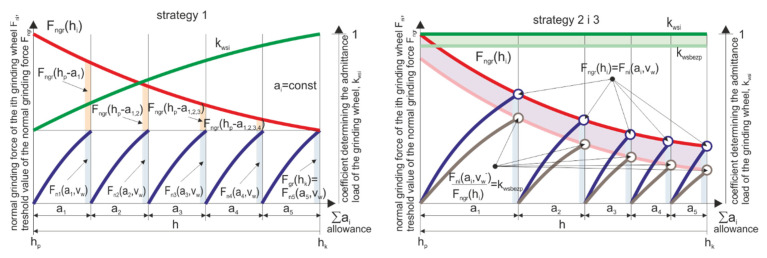
Loading of grinding wheels in the analyzed method of automatic machining of ceramic elements, taking into account three strategies for optimizing the distribution of the allowance.

The above optimization strategies lead to different loads on the grinding wheels involved in the machining (Figure 14).

Strategy 1 ensures equal wheel load. Unfortunately, the consequence of this is the adoption of the minimum possible table speed, and thus a significant reduction in the efficiency of the process. In addition, the last grinding wheel, which determines the accuracy of the shape and dimensions of the workpiece, works with the maximum load limit.

Strategy 2 ensures maximum process efficiency. The grinding wheels operate with the maximum permissible load. Uneven wear of grinding wheels working in series disturbs the optimal division of the allowance. This can be reduced by improving the selection of characteristics and the life of the individual wheels.

When selecting diamond grinding wheels, first of all, the data influencing the selection of the type, size and type of abrasive grains, abrasive concentration, type of bond, including the dynamic hardness of the grinding wheel, vibration damping ability, porosity and thermal conductivity were analyzed. As the size of the abrasive grains decreases, the occurrence of edge damage and structural damage to the workpiece surface gradually decreases. However, the grinding force and energy increase then, which means that the optimal grain size for each grinding wheel can be determined. By selecting the size of the abrasive grain and the concentration of the abrasive, the cutting potential, as well as the durability and life of the grinding wheel, can be influenced. A greater concentration of abrasive leads to an increase in the service life of the grinding wheel.

The basic expected feature of each binder is the ability to firmly hold the abrasive grains during the grinding process while ensuring a stable course of chipping and breaking the worn grains. Four basic types of binders are used in diamond grinding wheels: resin, ceramic, sintered metal and electroplated metal. Resin binders are characterized by high availability and a simple process of manufacturing grinding wheels, high susceptibility to shaping and good vibration damping in the grinding zone. These features determined the selection of the resin binder and the use of microaggregates. Ceramic binders are characterized by high suitability for grinding with constantly sharp blades, susceptibility to profiling, long retention of properties without the need for frequent dressing, but lower shape durability. Sintered metal binders are characterized by high durability, particular suitability for cutting and grinding narrow grooves, channels, high stiffness, good maintenance of the shape of the grinding wheel, good thermal conductivity (good heat dissipation from the grinding zone), potentially high suitability for high speed grinding, profiling operations are however more difficult, but the use of these binders compared to resins and ceramics means greater grinding forces and power. Electroplated metal binders are used in the form of layers of abrasive and a binder electroplated on the tool body, such grinding wheels can provide a higher concentration of abrasive, but the possibility of profiling is limited.

The surface topography of ceramic element after machining was performed by using a LEXT OLS 4000 (Olympus Co., Tokyo, Japan) confocal laser scanning microscope (CLSM).

The microscope used for the measurement was equipped with a dual confocal system, which used a beam of visible light with a wavelength of λ = 405 nm that was generated by a class II laser diode. Measurements were performed using an MPLAPON100XLEXT lens with a magnification of 2160× (field of view 128 μm, working distance 0.35 mm, numerical aperture 0.95).

The values of the roughness height parameters were determined for the measured surfaces in accordance with ISO 25178-2.

## 3. Results

### Research on Grinding Processes

The authors carried out many processes of grinding small ceramic elements using the developed automatic grinders. Tests of the power and moment of grinding of ceramic fittings (N 1500-rotors with a diameter of 7.4 mm) were carried out for the peripheral speed of the table *v_w_* = 5–20 mm/s, with gradations every 5 mm/s and for grinding allowances *a* = 0.05–0.2 mm in 0.05 mm increments. The purpose of the research was to compare the grinding process power with the use of: aggregate grinding wheels (grinding wheel with resin bond, hardness H and designation S 3020D-SD-160/125 M 75/BH, grinding wheels with hardness K and designation S 3020D-SD-160/125 M 75/BK) and a conventional grinding wheel (S 3020D-SD-160/125 M 75/BT).

**Figure 15:**
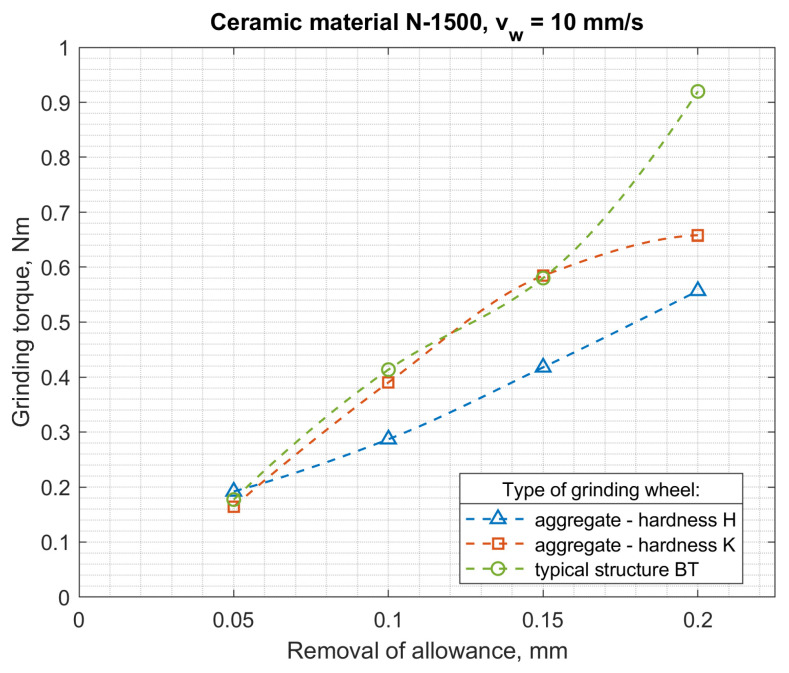
Dependence of the grinding torque on the allowance and the type of grinding wheel for the feed speed *v_w_* = 10 mm/s.

**Figure 16:**
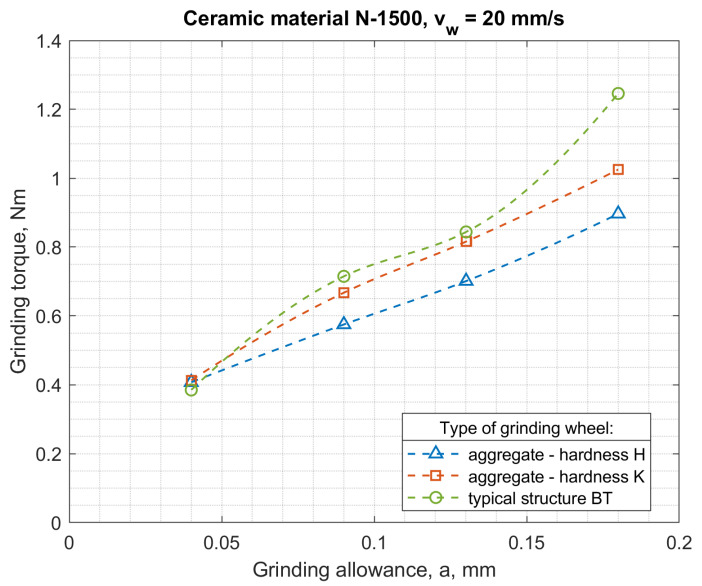
Dependence of the grinding torque on the allowance and the type of grinding wheel for the feed speed *v_w_* = 20 mm/s.

**Figure 17:**
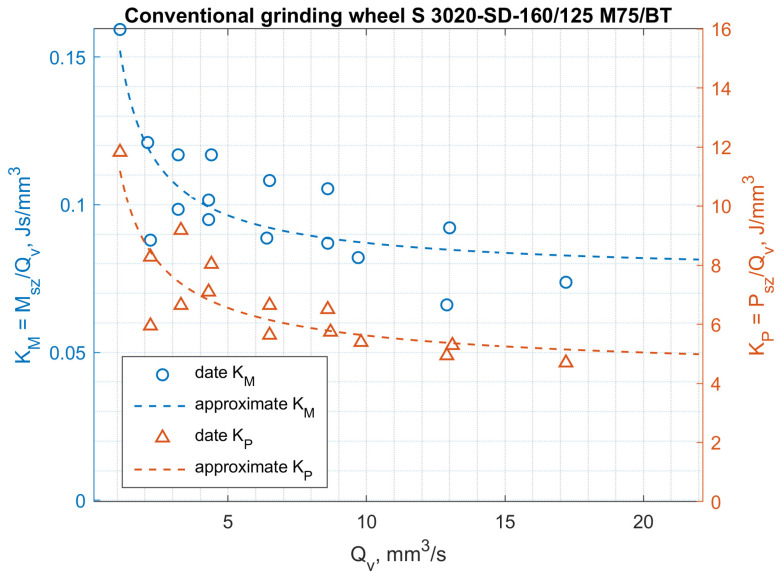
Influence of volumetric efficiency of grinding *Qv* on the *K_P_* and *K_M_* coefficients for a grinding wheel of conventional construction S 3020D-SD-160/125/M 75/BT.

**Figure 18:**
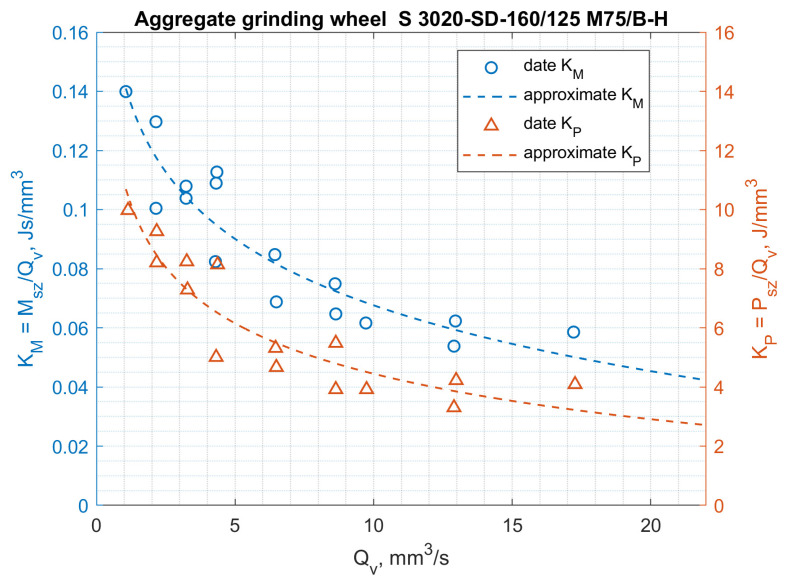
Influence of the volumetric efficiency of grinding *Qv* on the *K_P_* and *K_M_* coefficients for the aggregate grinding wheel S 3020D-SD-160/125/M 75/B-H.

The results of testing the power and torque of grinding ceramic fittings (rotors with a diameter of 7.4 mm) for maximally filled seats by a continuous stream of workpieces are shown in Figure 15 and Figure 16, and the results of testing the energy consumption of the process and the moment related to the grinding volumetric capacity are shown in Figure 17 and Figure 18.

The above results show that the thesis of lower specific grinding energy with the use of aggregate grinding wheels under the conditions of face machining with a hyperboloid (or conical) surface was confirmed for aggregate grinding wheels of low hardness. Rotor grinding has shown that the grinding power with the S 3020D-SD-160/125 M 75/BH grinding wheel during grinding with performance greater than 6 mm^3^/s was more than 30% lower than the grinding power with a conventional grinding wheel S 3020D-SD-160/125 M 75/BT. On the other hand, the use of an aggregate grinding wheel with higher hardness (K) S 3020D-SD-160/125 M 75/B-K allowed (in the range of grinding efficiency greater than 7 mm^3^/s) the power to be reduced by approx. 10%. For aggregate grinding wheels, a fourfold increase in the peripheral speed of the table caused an average 2.5-fold increase in grinding power, while for a conventional grinding wheel, the increase was over threefold. Aggregate grinding wheels under the conditions of the limitation imposed on the grinding power allowed higher grinding efficiency, although they contained a smaller mass of the diamond abrasive in their volume.

**Figure 19:**
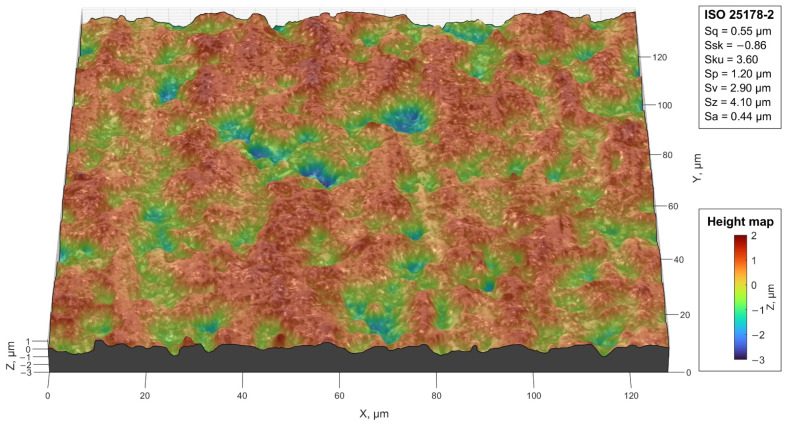
View of the surface topography and the values of 3D amplitude parameters for the ceramic surface after the machining process.

Figure 19 shows a view of a fragment of the ceramic surface topography after the machining process together with the values of the 3D amplitude parameters (ISO 25178-2). As a result of the measurement, the arithmetic mean surface height Sa = 0.44 µm and the maximum surface height Sz = 4.10 µm were obtained.

The life of the grinding wheel exceeded 64 h, and after this time the grinding wheel surface was renewed, without the need for correction of inclination angles. During the service life of the wheel, more than 76,000 items were machined.

**Figure 20:**
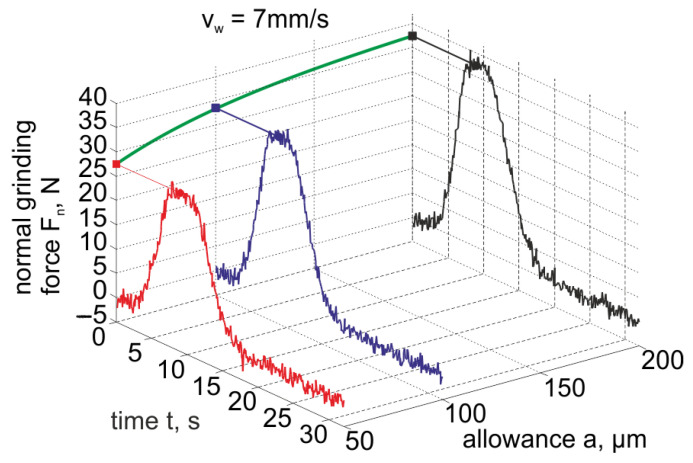
An example diagram of the normal grinding force component for the table travel speed *v_w_* = 7 mm/s when machining one ceramic element.

Subsequent research cycles concerned the grinding of ceramic sealing plates for valves, used, among others, for water batteries. As a result of the experiment, the values of the normal component of the grinding force *F_n_* and the vibration acceleration of the rotary table ${\dot{v}}_{s}$, the headstock assembly ${\dot{v}}_{w}$ and the grinding moment *M* were obtained as a function of the movement of the element through the grinding zone for various machining allowances. An example diagram of changes in the normal grinding force component during grinding of one ceramic element with the table speed *v_w_* = 7 mm/s is shown in Figure 20.

The results of the torque tests were used to verify the correctness of the obtained force components and the vibration acceleration of the grinding machine elements. The courses of changes in the acceleration of vibrations, due to the relatively high level of vibrations of the headstock assembly at idle speed, were not sensitive to changes in the size of the allowance in the case of grinding at low speeds. Therefore, in further analysis, only the assessment of changes in the components of grinding forces and the acceleration of vibrations of the rotary table was used.

Based on the research, it is shown that for monitoring the quality of products based on the deviation in height and flatness deviation, the values of the component of the normal grinding force and the vibration parameters of the headstock and the grinding table are useful.

The developed grinding method minimizes the appearance of edge fractures as a result of the entire surface adhesion of the machined elements to the grinding wheel surface and as a result of the decreasing speed of removal of the allowance (along the machining zone) from the value of 0.2 µm/mm to the zero value.

## 4. Conclusions

The publication presents the results of research on a new method and a device for automated grinding of small ceramic elements. It has been shown that the advantageous feature of the described method is multiple elongations of the machining zone (also for a small effective width of the grinding wheel surface) compared to face grinding with a chamfer wheel, and in comparison to grinding with the circumference of the grinding wheel, even several dozen times. This is achieved by the use of a face grinding with a hyperboloid active surface of the grinding wheel. The grinding zone extends from the zone where the path of the workpieces intersects with the outer contour of the grinding wheel to the zone where the workpieces extend beyond the contour of the active surface of the grinding wheel. For a given layer thickness of the allowance to be removed, it requires the selection of the values of the α and β angles of the grinding wheel axis inclination by one grinding wheel. As a result, a low stock removal rate and high uniformity of the local workpiece load in the grinding zone can be ensured. It is also advantageous that the machining marks gradually intersect as the workpieces are moved along the machining zone and the workpiece smoothly leaves the grinding zone.

An important feature of the method is the fixing of the workpieces with the use of the normal and tangential components of the grinding force as a result of placing the workpieces in shallow cuts in the profiled periphery. The objects rest on the flat surface of the table and are pressed against the low vertical wall of the profiled rim. In the described method, during the movement of the object along the grinding zone, several million grains are moved over its surface for each millimeter of the width of the processing zone. This means that, depending on the proportion of active grains, the allowance is removed by a much larger number of grains than in other grinding methods (for the fixed longitudinal feed rates of the workpiece and the rotational speed of the grinding wheel).

The developed method allows the direction of the machining traces to change into the machining zones, which ensures their favorable crossing. The use of innovative, special microaggregate grinding wheels enables the reduction of the grinding energy and increasing the shape durability of the resin-bonded grinding wheels with diamond micro-aggregates, in which small diamond abrasive grains are bonded with a metal bond.

The structural system for the microgrinding process with the use of the described method should enable precise setting of the α and β angles of the grinding wheel axis to remove small material allowances at the maximally extended grinding zone. Automation of the grinding process is possible thanks to self-clamping of workpieces in the machining zone, the long grinding zone, and sequential machining by a specified number of grinding wheels. This results in a machining cycle in which the workpieces pass through successive machining zones at one time, and the division of the allowance into individual grinding wheels can be made taking into account the features of the workpieces and the requirements for the machining results.

## Figures and Tables

**Table 1:** Composition of the processed ceramic elements.

Formula	CaCO_3_	TiO_2_	Parafin Emulsion	ZrO_2_	Other Admixtures
share	50%	40%	7%	1%	2%

**Table 2:** Features of grinding wheels with diamond microaggregates.

Aggregate Grinding Wheel Hardness	Size of Diamond Grains, µm	Concentration	Average Linear Aggregate Size *a_avg_*, µm	Average Distance between the Centers of the Aggregates *la_avg_*, µm	Percentage of Aggregates in the Volume of the Abrasive Layer, %	Unit Number of Aggregates, 1/cm^2^
H	160/125	75	540	1457.4	34.5	50
K	160/125	75	516	1444.1	33.2	50.4

**Table 3:** Features of diamonds in abrasive aggregates.

Size of Diamond Grains, µm	Number of Grains in One Carat	Concentration	Unit Volume Number of Grains *z*, 1/cm^3^	Unit Linear Number of Grains *z*1, 1/cm	Distance Between the Grains on the Active Surface *lz_avg_*, µm	Distance Between the Grains in the Volume of the Abrasive Layer *lzo_avg_*, µm
160/125	25,500	75	84,150	44	227	321

**Table 4:** Features of successive treatments in an automated microgrinding operation.

No.	Grain Size, µm	Angle α, °	The Height of the Element after Processing, mm	Removal of Allowance, mm
1.	160–125	0°35′	1.57	0.03–0.23
2.	125–100	0°35′	1.35	0.22
3.	125–100	0°35′	1.13	0.22
4.	100–80	0°25′	1.05	0.08
5.	63–50	0°	1.00	0.05

**Table 5:** Values characterizing the automatic device for grinding small ceramic elements for design solutions for the processing of ceramic capacitor elements.

Parameter	Value
Diameter of ground elements	7.4_−0.2_ mm
Height of elements after grinding	0.98_±0.02_ mm
Machining efficiency—elements/h	1280–6800
Table rotation speed	0.066–0.175 min^−1^
Table diameter	1060 mm
Speed of moving objects	4–20 mm/s
Number of locating sockets	324
Number of grinding headstocks	5
Spindle speed	2820 min^−1^
Diameter of the diamond grinding wheels	200 mm
Vertical spindle travel	up to 10 mm
Elementary plot on the scale of vertical spindle travel	0.001 µm
Compressed air supply pressure	min. 0.2 MPa
Compressed air flow rate	10 m^3^/min
Water supply pressure	min. 0.2 MPa
Water flow rate	3 dm^3^/min
Supply voltage/frequency	3 × 380 V/50 Hz
Drive motor power of each grinding wheel	0.75 kW
Table drive motor power	0.37 kW
Total, installed power	4.48 kW
Overall dimensions A × B × H	1500 mm × 1700 mm × 1290 mm
The mass of the device	about 1100 kg

**Table 6:** The results of optimization of the allocation of the allowance leading to the determination of safe machining parameters and monitored process features.

Headstock Number	Safe Parameters	Element with a Height		
		Minimal (*a_c_* = 100 µm)	Average (*a_c_* = 200 µm)	Maximal (*a_c_* = 300 µm)
1–3	*v_w_*, mm/s	8.5	5.9	4.4
1	*a*_1_, µm	64.2	103.7	139.1
	*F_n_*_1_, N	32.5	31.4	30.4
2	*a*_2_, µm	29.7	67.5	100.6
	*F_n_*_2_, N	29.8	29.9	28.7
3	*a*_3_, µm	6.1	28.7	60.3
	*F_n_*_3_, N	22.3	23.3	24.1

## Data Availability

Data sharing is not applicable to this article.
